# Polycomb Group Protein Pcgf6 Acts as a Master Regulator to Maintain Embryonic Stem Cell Identity

**DOI:** 10.1038/srep26899

**Published:** 2016-06-01

**Authors:** Chao-Shun Yang, Kung-Yen Chang, Jason Dang, Tariq M. Rana

**Affiliations:** 1 Department of Pediatrics, University of California San Diego School of Medicine, 9500 Gilman Drive MC 0762, La Jolla, California, 92093, USA; 2 Department of Bioengineering, University of California San Diego, La Jolla, California, 92093, USA.; 3 Institute for Genomic Medicine and Moores Cancer Center, University of California San Diego School of Medicine, 9500 Gilman Drive, La Jolla, California, 92093, USA.

## Abstract

The polycomb repressive complex 1 (PRC1) is a multi-subunit complex that plays critical roles in the epigenetic modulation of gene expression. Here, we show that the PRC1 component polycomb group ring finger 6 (Pcgf6) is required to maintain embryonic stem cell (ESC) identity. In contrast to canonical PRC1, Pcgf6 acts as a positive regulator of transcription and binds predominantly to promoters bearing active chromatin marks. *Pcgf6* is expressed at high levels in ESCs, and knockdown reduces the expression of the core ESC regulators *Oct4*, *Sox2*, and *Nanog*. Conversely, *Pcgf6* overexpression prevents downregulation of these factors and impairs differentiation. In addition, Pcgf6 enhanced reprogramming in both mouse and human somatic cells. The genomic binding profile of Pcgf6 is highly similar to that of trithorax group proteins, but not of PRC1 or PRC2 complexes, suggesting that Pcgf6 functions atypically in ESCs. Our data reveal novel roles for Pcgf6 in directly regulating *Oct4*, *Nanog*, *Sox2*, and *Lin28* expression to maintain ESC identity.

The two key characteristics of embryonic stem cells (ESCs) are self-renewal and pluripotency^1^,^2^. Several transcription factors, including Oct4, Sox2, and Nanog, are components of a complex transcriptional network of positive and negative feedback loops that maintain ESC identity while repressing the expression of genes that promote differentiation^3^,^4^. Previous studies have established the importance of epigenetic modulation of gene expression in ESCs, including the chromatin-modifying polycomb group (PcG) and trithorax group (TrxG) proteins, which predominantly mediate gene repression and activation, respectively^5^,^6^.

PcG proteins were first identified as transcriptional repressors of the Hox gene cluster that regulates body segmentation in *Drosophila melanogaster*^7^. Since then, PcG proteins have been shown to play crucial roles in many physiological processes, including stem cell maintenance, cell fate specification, and cycle control, as well as in pathological processes such as cancer development^8^,^9^,^10^. PcGs exist as two multimeric protein complexes; polycomb repressive complex 1 (PRC1) and 2 (PRC2), both of which function as gene silencers. In mammalian cells, multiple PRC1 complexes are formed through combinatorial association of 5 core proteins: Cbx (Cbx2, 4, 6, 7, and 8), Pcgf (Pcgf1, 2, 3, 4, 5, 6), Phc (Phc1, 2, and 3), Ring finger proteins (Ring1/Ring1b or Rnf1/Rnf2), and Rybp^11^. The catalytic subunits, Ring1 and Rnf2, are E3 ubiquitin ligases that conjugate a single ubiquitin molecule to lysine 119 of histone H2A (H2AK119ub1)^12^,^13^, a repressive histone mark associated to preventing RNA polymerase II transcriptional elongation^14^. The major components of mammalian PCR2 are Suz12, Ezh, Eed, Rbbp4, and Rbbp6. In PRC2 complexes, the primary catalytic subunit is the histone methylase Ezh, which di- or tri-methylates histone H3K27 to mark transcriptionally silent genes^9^,^15^,^16^. Similarly, TrxG proteins regulate numerous genes expression states through their activities in methylating/acetylating histones and remodeling chromatin^17^. In mammals, great diversity of TrxG proteins has been shown, including Mll2, Ash2l, Rbbp5, and Wdr5^17^. In contrast to PRC1/2, TrxG complexes maintain active chromatin states by acetylation of H3K27 and methylation of H3K4^17^. Therefore, PcG proteins function antagonistically to TrxG proteins to maintain homeostatic status of gene expression in various biological processes.

PRC1 and PRC2 are proposed to act sequentially to repress transcription. In this model, chromatin methylation by PRC2 serves to recruit PRC1, which in turn deposits H2AK119u1 and enhances gene repression^18^,^19^,^20^,^21^,^22^. Interestingly, recent findings suggest that PRC1-dependent recruitment of PRC2 forms a positive feedback loop establishing repressive chromatin domains^23^,^24^. Furthermore, the combinatorial complexity and composition of PRC1 suggests that these complexes may have more diverse functions than first thought; a possibility that is supported by an increasing body of evidence^25^,^26^,^27^,^28^. For example, proteomic profiling of PRC1 components has revealed the existence of at least 4 major sub-complexes defined by the 6 Pcgf proteins, each of which has unique chromatin-binding sites^26^. Furthermore, Rybp and Cbx form mutually exclusive complexes, and only Rybp-PRC1 complexes show prominent ubiquitin ligase activity^26^. Interestingly, recruitment of Rybp-PRC1 complexes to chromatin is independent of PRC2 and H3K27me3^29^, strengthening the probability that PRC1 complexes and/or components may have functions beyond gene repression. A number of studies have shown that PRC1 complexes play important roles in ESCs. PRC1 complexes are required to maintain ESC status by inhibiting several differentiation genes, including the Hox gene family^30^. The 5 Cbx homolog proteins have been shown to confer nonoverlapping target specificity on PRC1 complexes^27^, resulting in repression of unique gene sets during ESC self-renewal and differentiation^25^. Recently, a noncanonical L3mbtl2-containing PRC1 complex was shown to display atypical binding activity that correlates with ESC proliferation and early differentiation^31^. Notably, Pcgf6, Wdr5, and L3mbtl2 reside in the same atypical PRC1 complex^26^. Wdr5 is also one of the main components in TrxG complexes and has been reported to play important roles in ESC gene expression^32^. Pcgf6 has been shown to be required to maintain ESC self-renewal by an RNAi screen^33^ and to be the major Posterior sex combs (Psc) component in Cbx7-bound PRC1 complexes in ESCs^34^. In a recent study, Pcgf6 was identified as a key regulator of mouse ESC pluripotency and self renewal, iPS reprogramming and mesodermal differentiation^35^. Knockdown of Pcgf6 was shown to decrease expression of core ESC transcription factors Oct4, Sox2 and Nanog, reduce cellular reprogramming and increase mesodermal and testes-specific gene expression^35^. Pcgf6 can replace Sox2 in the mouse somatic reprogramming process to generate germline-competent iPS cells^35^. In all, Pcgf6 has been shown to play a repressive role in modulating mesodermal-specific lineage genes, maintaining embryonic stem cell pluripotency and facilitating iPSC reprogramming.

In our recent work, we utilized an unbiased genome-wide RNAi screen and integrative transcriptome analysis to dissect the determinants required for the induced reprogramming process^36^. Consistent with previous works, we found that Pcgf6 is a positive regulator of ESC self-renewal. However, it remains unclear how Pcgf6 modulates the mRNA expression level of core ESC transcriptional regulators (e.g. Oct4, Sox2, Nanog). Therefore, this prompted us to further investigate the functions of Pcgf6 in ESC self-renewal and differentiation.

Here, we show that Pcgf6 is essential for sustaining the expression of ESC-specific genes, including *Oct4, Sox2, Klf4, Nanog*, and *Lin28*, and is required to maintain ESC identity and to support efficient induced reprogramming of both mouse and human somatic cells. Moreover, overexpression of Pcgf6 suppresses differentiation of ESCs, consistent with its ability to maintain high levels of *Oct4, Sox2*, and *Nanog* expression. Surprisingly, and in contrast to the canonical action of PRC1 complexes, Pcgf6 is bound to chromatin predominantly at active genes and shows a binding specificity more similar to that of TrxG than either PRC1 or PRC2. Finally, our data show that Pcgf6 directly modulates the expression of *Oct4, Sox2, Nanog*, and *Lin28* to promote the maintenance of ESC identity.

## Results

### Pcgf6 is Required to Maintain ESC Identity

**Figure 1:**
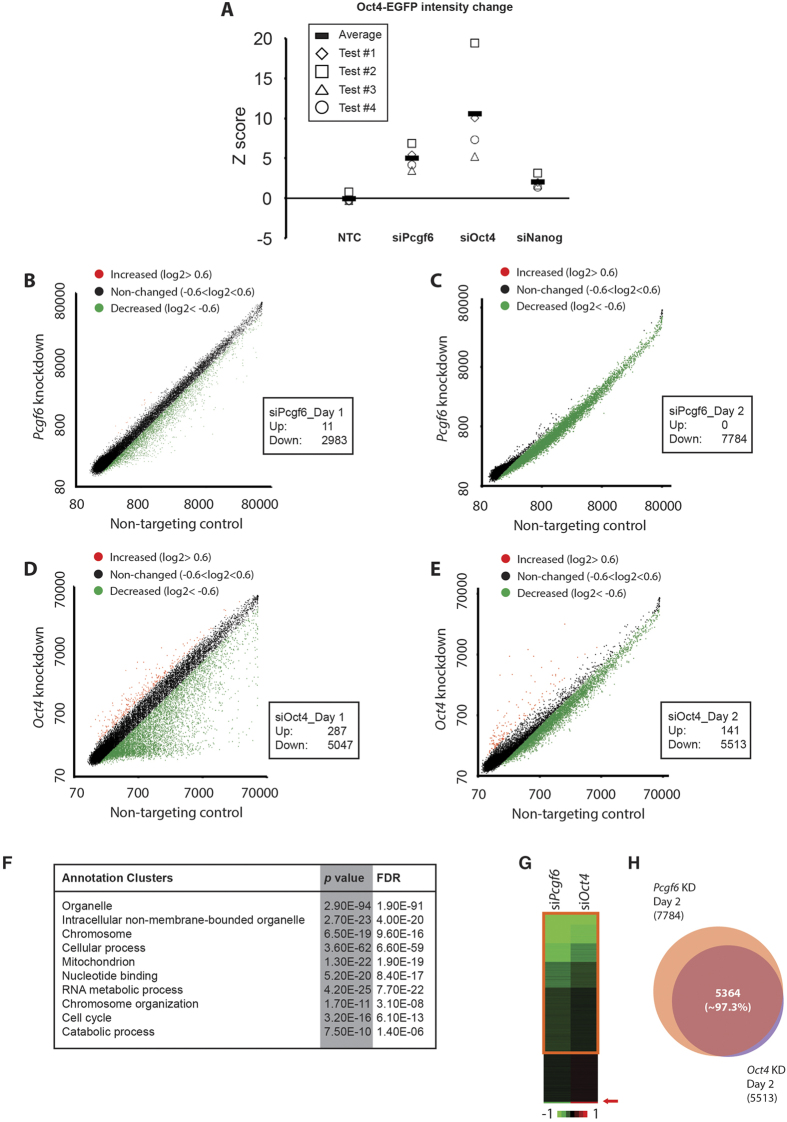
Pcgf6 is Essential for the Maintenance of ESC Identity. (**A**) Distribution plot showing z score of Oct4-EGFP signal changes with various siRNA treatments. Oct4-EGFP ESCs were transfected with indicated siRNAs (si*Pcgf6*, si*Oct4*, si*Nanog*) and EGFP signal was detected and measured by flow cytometry. Non-targeting control siRNA serves as negative control (NTC). Z score was calculated by normalizing to NTC. (**B~E**) Scatter plot showing mRNA expression changes in *Pcgf6-* or *Oct4-*depleted CCE ESCs one or two days after siRNA transfection. Cells were transfected with *Pcgf6* or control siRNAs for 3–5 h, and mRNA expression was analyzed by microarray 1 or 2 days later. Green dots indicate genes showing reduced expression (<−0.6 on log_2_ scale) in *Pcgf6*- or *Oct4-*depleted compared with control cells. Red dots indicate increased genes (>0.6 log_2_) and black dots indicate non-differentially-expressed genes with *Pcgf6* or *Oct4* knockdown. The number of differentially expressed genes is shown in the box in each panel. (**F**) Table showing gene ontology analysis (GO) of decreased genes in *Pcgf6*-depleted ESCs. 2983 decreased genes at day 1 post *Pcgf6*-depletion in ESCs were analyzed using DAVID^54^,^55^. Probability is represented as Score (−log_10_ *p*-value). (**G**) Heat map showing mRNA expression profiling in CCE ESCs depleted of *Pcgf6* or *Oct4*. Cells were transfected with *Oct4*, *Pcgf6,* or control siRNAs for 3–5 h, and mRNA expression was analyzed by microarray 1 day later. Results in *Oct4* and *Pcgf6* knockdown (KD) cells were normalized to cells treated with control siRNA and then subjected to K-mean clustering analysis. Color bar is on a log_2_ scale. Red arrow indicates the small proportion of genes showing increased expression in *Oct4* KD cells, but which are decreased in *Pcgf6* KD cells. (**H**) Venn diagram showing overlap between genes suppressed by *Pcgf6* or *Oct4* KD. mRNA expression was analyzed by microarray 2 days after transfection of CCE ESCs with siRNA, and results were normalized to cells treated with control siRNA. The data show that 97.3% of genes that were reduced by *Oct4* KD were also reduced by *Pcgf6* KD.

To determine whether Pcgf6 plays a role in modulating ESC identity, we examined the effects of *Pcgf6* knockdown (KD) in Oct4-EGFP mouse ESCs. Oct4-EGFP ESCs were transfected with non-targeting control (NTC) or *Pcgf6*-specific siRNA for 3–5 h, and EGFP signal was detected four days later by flow cytometry. Four independent experiments (test #1~4) were conducted and the percentage of EGFP signal in each siRNA-treated sample was measured (Fig. 1A). Change of Oct4-EGFP signal was converted into Z score by normalizing to NTC treated samples (Fig. 1A). We consistently observed that *Pcgf6* depletion in ESCs caused Oct4-EGFP decrease. In addition to Oct4-EGPF ESCs, CCE ESCs were also employed and treated with specific siRNAs targeting *Pcgf6*, *Oct4*, or non-targeting control (Control). Cell morphology and Oct4-EGFP expression was examined 4 days later. Whereas Oct4-EGFP ESCs treated with control siRNA retained the typical colony morphology of ESCs and expressed high levels of Oct4 (EGFP signal), we observed that *Pcgf6* KD induced differentiation, as indicated by the flat cell morphology and concomitant loss of Oct4-EGFP expression (Figure S1A). mRNA knockdown efficiency was examined by RT-qPCR at day 4 post siRNA transfection (Figure S1B). We further examined *Oct4* expression by using four shRNA constructs and four siRNAs (Figure S1C,D), showing specificity of *Pcgf6* depletion in our assays.

Because other PRC1 components (e.g., Ring1b and Cbx7 25,30) have been shown to maintain ESC self-renewal by repressing differentiation genes, we next performed transcriptome analysis of CCE ESCs following *Pcgf6* KD. To minimize indirect or confounding effects caused by the loss of *Oct4* expression in *Pcgf6* KD cells (Figs 1A, S1C, S1D), we performed mRNA expression profiling one day (Fig. 1B,D) and two day (Fig. 1C,E, Table S1) after treatment with non-targeting control, *Oct4*, or *Pcgf6* siRNA. We found that *Pcgf6* KD had a marked effect on gene expression in these cells, but interestingly, the majority of differentially expressed genes were downregulated (Fig. 1B,C), in contrast to the de-repression observed in cells depleted of other PRC1 components^30^. With *Pcgf6* depletion with siRNA in ESCs, gene ontology analysis shows the top 10 over-representative cellular regulations involved in different functions, indicating functional diversity of Pcgf6 (Fig. 1F). Notably, the downregulated genes included key factors of the ESC core circuitry, *Oct4*, *Sox2*, *Klf4,* and *Nanog*, which were reduced 50–70% by *Pcgf6* KD (Figure S1D). These data demonstrate that Pcgf6 is required to maintain an ESC-specific transcriptome, and further suggest that Pcgf6 might act through a mechanism additional to the canonical PRC1 pathway in ESCs.

We next investigated whether Pcgf6 and Oct4 regulate the similar population of genes in ESCs by examining the transcriptome of CCE ESCs treated with control, *Oct4*, or *Pcgf6* siRNA in Fig. 1B,D. Many genes were similarly downregulated in both *Pcgf6* and *Oct4* KD ESCs (Fig. 1G, orange rectangle); however, we also detected a small subset of genes that were specifically increased only in *Oct4*-depleted ESCs (red arrow in Fig. 1G), suggesting that Pcgf6 and Oct4 have largely overlapping but not identical target genes. Consistent with this, decreased genes at day 2 have strikingly high overlapping ratio (~97.3%) in both *Oct4* KD and *Pcgf6* KD cells (Fig. 1H). We confirmed that these effects were not limited to a single siRNA by examining the expression of a subset of genes in CCE ESCs treated with 4 different *Pcgf6*-specific siRNAs (Figure S1D). All 4 siRNAs reduced the expression of a panel of target genes, albeit to slightly varying degrees (Figure S1D). Many differentially expressed genes (400 out of 892) and cellular functions with *Oct4* knockdown in previous report^32^ are consistently identified in *Oct4*-depleted ESCs in this study (Figure S1E,F). Collectively, our data demonstrate that Pcgf6 plays an essential role in maintaining the expression level of a large number of genes in ESCs, including the core regulators (*Oct4, Sox2, Nanog, Klf4*) that preserve ESC identity.

### Pcgf6 Promotes the Expression of *Oct4*, *Sox2*, and *Nanog* and Suppresses Differentiation

Having shown that Pcgf6 is critical for maintaining ESC identity, we next asked whether overexpression of Pcgf6 affects ESC differentiation. For this, we derived several CCE ESC clones stably overexpressing Pcgf6 (termed Pcgf6-mESCs). The clones were confirmed to express high levels of Pcgf6 protein (Figure S1G) and to exhibit normal ESC morphology with dome-shaped colonies (Figure S1H). Four clones (#1, 4, 6, 7) were selected for further analysis, and CCE ESCs transfected with EGFP were used as controls (Figure S1H).

**Figure 2:**
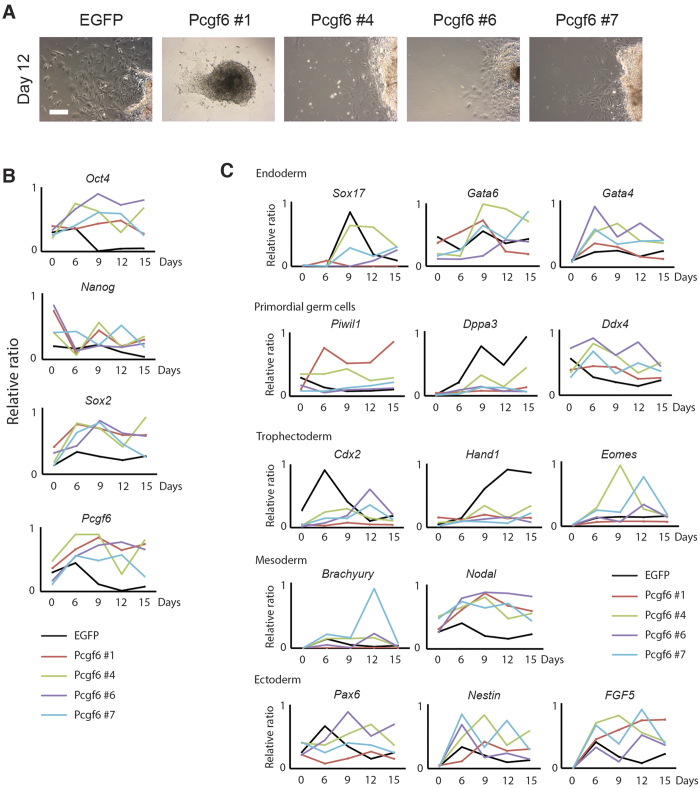
Pcgf6 Regulates the Core Circuitry and Maintains Pluripotency in ESCs. (**A**) Micrographs showing abnormal EB morphology of differentiating Pcgf6-mESCs. CCE ESCs were stably transfected with Pcgf6 or EGFP (control), and *in vitro* differentiation was assessed by monitoring EB formation over 15 days. Images were taken using phase contrast microscopy from day 12 cultures of control cells and 4 individual clones overexpressing Pcgf6. Scale bar = 100 μm. (**B,C**) Expression of ESC-specific (**B**) and differentiation marker (**C**) genes during *in vitro* differentiation of EGFP- or Pcgf6-overexpressing CCE ESCs. mRNA levels were measured on days 0, 3, 6, 9, 12, and 15 and are presented as the ratio of mRNA levels relative to the highest expression level during differentiation. Four biological replicates are shown in four different color lines and control (EGFP-overexpressing CCE) is in black dotted line.

To determine how Pcgf6 overexpression affects ESC pluripotency, we examined the expression of differentiation markers during embryoid body (EB) formation *in vitro*. As expected, control EGFP-mESCs showed robust differentiation and had migrated extensively by 12 days of culture (Fig. 2A). In sharp contrast, all 4 Pcgf6-mESC clones remained in relatively compact colonies with only limited extension (Fig. 2A), suggesting that Pcgf6 overexpression perturbs *in vitro* spontaneous differentiation. To confirm this, we measured mRNA levels of ESC-specific genes and a panel of 14 genes specifically expressed in different tissue types. Consistent with their aberrant EB formation, the Pcgf6-mESCs clones maintained high levels of *Oct4, Sox2,* and *Nanog* mRNA over the 15 days of culture (Fig. 2B), demonstrated severe misregulation of the differentiation marker genes in comparison to the control cells (Fig. 2C). These data show that Pcgf6 overexpression impairs spontaneous ESC differentiation *in vitro*, likely by preserving expression of high levels of *Oct4, Sox2*, and *Nanog*, which in turn disturb the differentiation process.

### Pcgf6 is Required for Efficient Reprogramming of Somatic Cells

**Figure 3:**
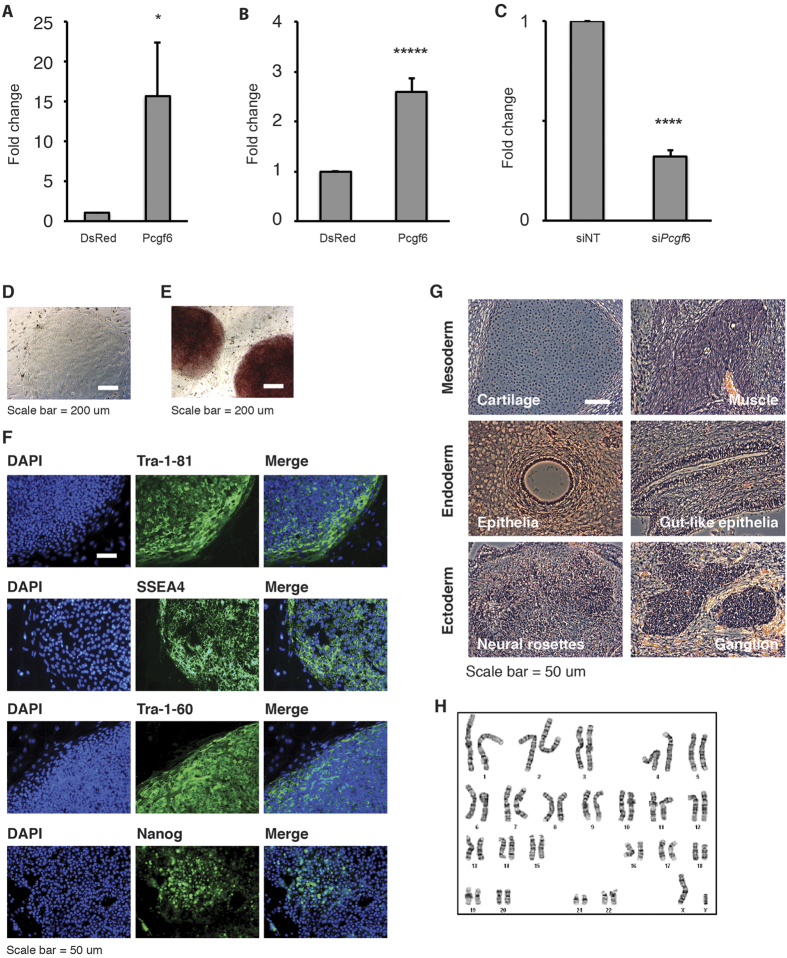
Pcgf6 Enhances Induced Reprogramming Efficiency. (**A**) Reprogramming efficiency of Oct4-EGFP MEFs transduced with OSKM and either Pcgf6 or DsRed (control) retroviruses. EGFP+ colonies were counted 2 weeks post-transduction, and the results are expressed as colony number relative to control DsRed-miPSCs. Results are the mean ± SEM of n ≥ 5. *p < 0.05 by Student’s t-test. (**B**) Reprogramming efficiency of human BJ fibroblasts transduced with OSKM and either Pcgf6 or DsRed (control) retroviruses. AP+ colonies were counted 3–4 weeks post-transduction, and the results are expressed as for (**A**). Results are the mean ± SEM of n ≥ 5. *****p < 0.000005 by Student’s t-test. (**C**) Reprogramming efficiency of Oct4-EGFP MEFs depleted of *Pcgf6*. Cells were treated with non-targeting (siNT) or Pcgf6-specific siRNA for 3–5 h and then transduced with OSKM retroviruses. EGFP+ colonies were counted 2 weeks later, and the results are expressed as for (**A**). Results are the mean ± SEM of 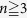. ****p < 0.00005 by Student’s t-test. (**D–F**) Images of Pcgf6-hiPSCs derived as in (**B**). Cells were cultured on feeders for at least 4 passages before imaging of: cell morphology (**D**), AP staining (**E**), and Tra-1-81, SSEA4, Tra-1-60, and Nanog immunofluorescence staining (**F**). Scale bars = 200 μm (**D,E**) or 50 μm (**F**). (**G**) Histological staining of mesodermal, endodermal, and ectodermal tissues in teratomas formed from Pcgf6-hiPSCs. Cells were injected under the kidney capsule of SCID mice, and teratomas were removed and analyzed ~10 weeks later. Scale bar = 50 μm. Images were taken using either phase contrast microscopy or fluorescent microscopy. (**H**) Normal karyotype of Pcgf6-hiPSCs. Cells were cultured for ~2 months on feeder cells before karyotype analysis. Chromosomes in the metaphase of 20 cells were counted, and 4 cells were examined for G-band staining with band resolution at 450–525. All cells contained normal karyotypes with 46 chromosomes including XY, and no clonal abnormalities were detected.

The finding that Pcgf6 regulates the expression of *Oct4, Sox2*, and *Nanog* in ESCs suggests that it may also influence induced reprogramming of human and mouse somatic cells. To test this, we simultaneously transduced the human fibroblast cell line BJ or Oct4-EGFP MEFs with OSKM and either Pcgf6 or the red fluorescent protein DsRed (control). Pcgf6 was confirmed to localize to the nucleus in these cells (Figure S1I), indicating proper biological function of Pcgf6 proteins. Two weeks after induction, the reprogrammed cells (termed Pcgf6-miPSCs and Pcgf6-hiPSCs) were detected as EGFP+ (miPSCs) or AP+ (hiPSCs) colonies. Overexpression of Pcgf6 had a dramatic effect on MEF reprogramming, increasing the number of colonies by ~15-fold compared with colonies derived from control MEFs (Fig. 3A). The other beneficial effect of Pcgf6 has been also shown to replace Sox2 in reprogramming process in a recent report^35^. Similarly, overexpression of Pcgf6 improved the reprogramming efficiency of human BJ fibroblasts by ~2.5-fold (Fig. 3B). Conversely, depletion of Pcgf6 with siRNAs greatly compromised the induced reprogramming of Oct4-EGFP MEFs (Fig. 3C), confirming that Pcgf6 is required for efficient somatic cell reprogramming.

To determine whether the Pcgf6-iPSCs retain the biological properties of authentic ESCs, we picked Pcgf6-iPSCs and examined the ESC-specific gene expression, *in vivo* pluripotency, and karyotypes of the reprogrammed cells. Endogenous Oct4 expression was clearly reactivated in Pcgf6-miPSCs (Figure S1J) and teratomas formed 3 weeks after injection of Pcgf6-miPSCs into nude mice contained diverse tissue types, including epidermal cells, epithelial cells, muscle cells, and neural rosettes (Figure S1K). Similarly, Pcgf6-hiPSCs had acquired functional ESC identity, as evidenced by the characteristic flat colonies, strong AP staining, and positive immunostaining of the pluripotency markers Tra-1-81, SSEA4, Tra-1-60, and Nanog (Fig. 3D–F). Moreover, Pcgf6-hiPSCs also acquired pluripotency, as demonstrated by the formation of teratomas containing tissues from all three germ layers at 10 weeks after injection into SCID mice (Fig. 3G). Importantly, we found no evidence of aberrant karyotypes in Pcgf6-hiPSC clones (Fig. 3H). Collectively, these results demonstrate that Pcgf6 is required for efficient reprogramming and that Pcgf6-derived iPSCs acquired fully pluripotent state.

### ChIP-Seq Analysis Reveals the Genomic Binding Profile of Pcgf6

**Figure 4:**
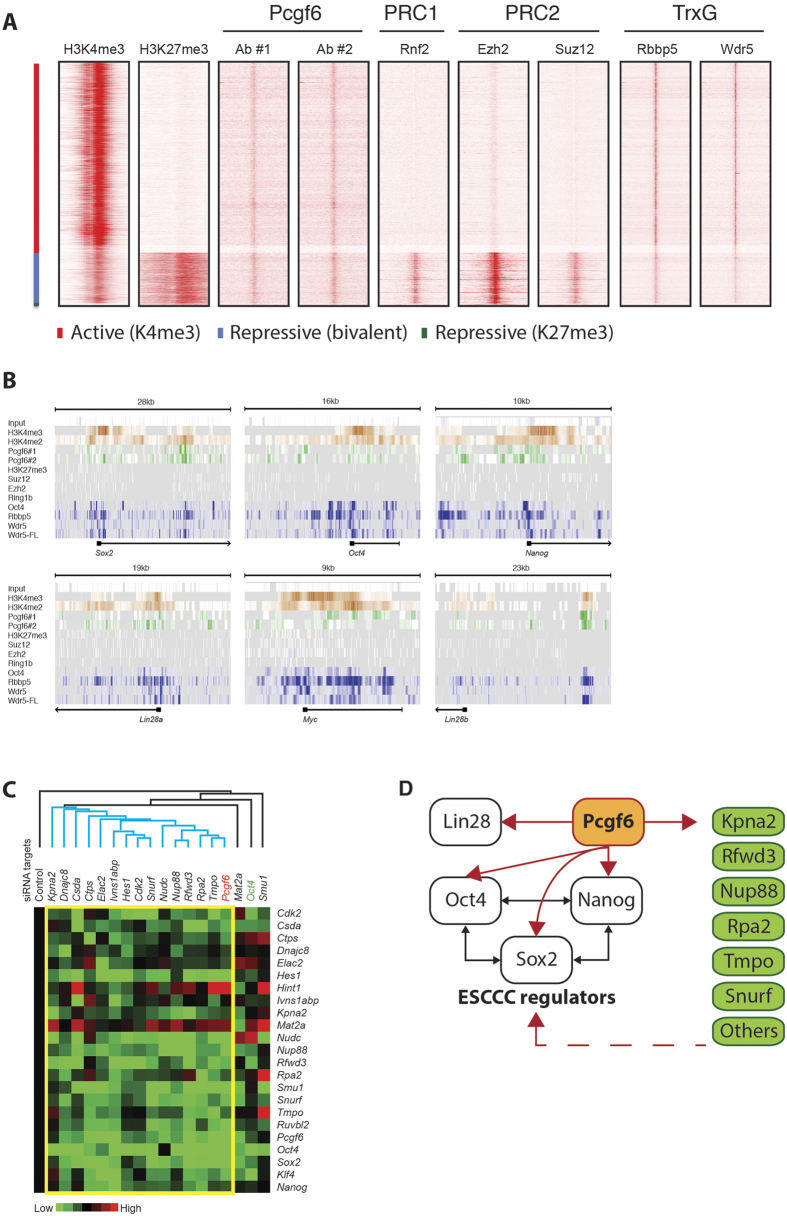
Pcgf6 Directly Regulates Key Pluripotency Factors in ESCs, Including Oct4, Sox2, Lin28b, and Nanog. (**A**) Heat map representations showing the chromatin-binding distribution of various epigenetic regulators in CCE ESCs. For Pcgf6, ChIP-seq experiments were performed with two different antibodies, as indicated (Ab#1 and 2; See Methods). ChIP-seq data for H3K4me3, H3K27me3, Suz12, Ezh2, and Rnf2 were obtained from the Bernstein lab^38^, and data for Rbbp5 and Wdr5 were obtained from the Ihor lab^32^. Regions decorated with active (H3K4me3), repressive (H3K27me3), and bivalent poised/repressive (both H3K4me3 and H3K27me3) chromatin marks are indicated by the vertical bar at left. (**B**) Pcgf6 binds promoter regions of key pluripotency genes in ESCs. ChIP-seq data for Pcgf6 (this study) were uploaded to the Integrative Genomics Viewer browser and compared with ChIP-seq data for H3K27me3, Suz12, Ezh2, and Rnf2 (as above) and Oct4, Rbbp5, Wdr5, and Wdr5-FL (Ihor lab^32^). The scale of each genomic region is indicated at the top of each panel. The TSSs of *Sox2, Oct4, Nanog, Lin28a, Myc*, and *Lin28b* target genes are shown as black squares with the arrow showing transcription direction. Green indicates Pcgf6 binding regions; orange indicates regions with histone modification markers; blue indicates regions bound by Oct4, PRC1, PRC2, and TrxG components. (**C**) Heat map showing that changes in gene expression caused by Pcgf6 knockdown is recapitulated by knockdown of Pcgf6 targets. CCE ESCs were transfected with siRNAs targeting various Pcgf6-bound genes (named at the top), and RT-qPCR was performed ~24 h later to detect expression of a panel of Pcgf6-bound genes (listed at right). Expression levels were normalized to cells treated with the non-targeting siRNA (Control) and displayed as a heat map. The hierarchical tree was created by Cluster and visualized by Java TreeView. The blue hierarchical lines and yellow rectangle indicate the major cluster. (**D**) Proposed model for the central role of Pcgf6 in modulating the ESC core circuitry. Pcgf6 activates the key regulators Oct4, Sox2, Nanog, and Lin28 in ESCs in a noncanonical fashion and directly regulates the expression of other factors, including *Kpna2, Rfwd3, Nup88, Rpa2, Tmpo*, and *Snurf*, which positively regulate the ESC core circuitry.

To investigate the mechanisms by which Pcgf6 regulates gene expression in ESCs, we performed chromatin immunoprecipitation with massively parallel DNA sequencing analysis (ChIP-seq) on CCE ESCs using two independent Pcgf6-specific antibodies. The specificity of antibodies was confirmed by western blot assay (Figure S2A). The ChIP-seq data were processed and visualized as heat maps after normalization against input DNA. We compared the Pcgf6 data (Table S2) with published ChIP-seq datasets for H3K4me3 and H3K27me3^37^,^38^ to determine whether Pcgf6 bound preferentially at sites of actively transcribed (H3K4me3), repressed (H3K27me3), or poised/repressed (H3K4me3 and H3K27me3) genes. In parallel, we compared ChIP-seq data^32^,^38^ for the PRC1 component Rnf2, the PRC2 components Ezh2 and Suz12, and two TrxG proteins, Rbbp5 and Wdr5 (Fig. 4A). As expected, the PRC1 and PRC2 components Rnf2, Ezh2, and Suz12 bound pervasively to promoters with repressive H3K27me3 or bivalent marks, whereas the TrxG proteins bound predominantly to sites bearing the active H3K4me3 mark (Fig. 4A). Notably, the majority of Pcgf6-binding sites colocalized with active H3K4me3-marked promoters (Fig. 4A), which is consistent with the widespread reduction in gene expression in *Pcgf6*-depleted mESCs (Fig. 1B,C). Indeed, of the Pcgf6-bound sites colocalizing with chromatin marks, ~75% were marked by H3K4me3 alone, 23% by both H3K4me3 and H3K27me3, and only 2% by H3K27me3 alone (Figs 4A and S2B,S2C). These findings strongly suggest that Pcgf6-containing complexes are directly involved in gene activation, which stands in stark contrast to the canonical role of PRC1 complexes in gene repression. To probe this further, we examined the degree of overlap in Pcgf6-bound and Rnf2-bound target sites and found that less than 14% of Pcgf6-bound targets were also bound by Rnf2 (Figure S2D). In addition, only ~3% of Pcgf6 were marked with H2AK119u1 (Figure S2D). These data suggest that the majority of Pcgf6-bound sites in ESCs are not associated with Rnf2- and/or Rybp-containing PRC1 complexes. To test this, we separated CCE ESC macromolecular complexes by fast protein liquid chromatography (FPLC) and examined the Pcgf6 and Rnf2 content of the resulting fractions by western blotting. We found that the peaks of Pcgf6-containing complexes are different from those of PRC1 (Rnf2) (Figure S2E), showing that Pcgf6 resides in different complexes from canonical PRC1. Collectively, these findings demonstrate that Pcgf6 binds predominantly at active promoters that are not ubiquitinated or bound by Rnf2, suggesting that Pcgf6 may have atypical functions in ESCs.

### Pcgf6 Acts as a Master Regulator to Modulate Oct4, Sox2, and Nanog Expression

Because the large-scale ChIP-seq analyses indicated that the majority of loci bound by Pcgf6 in ESCs were not bound by PRC2 or canonical PRC1 complexes, we next examined the promoters of a number of genes important for ESCs more closely. Components of both PRC1 and PRC2 complexes (Suz12, Ezh2, and Rnf2) were detected at the promoter region of the Hox gene cluster, which was expected given their role in repression of these genes (Figure S3A). Pcgf6 also bound to the Hox promoters, as did Oct4 and the TrxG proteins Rbbp5, Wdr5, Wdr5-FL (Figure S3A), showing that differentiation genes are marked as bivalent regions by both PRC1/2 and TrxG complexes. We also examined the binding profiles of Pcgf6-bound target genes. For examples, we selected hes family bHLH transcription factor 1 (Hes1), methionine adenosyltransferase II alpha (Mat2a), and karyopherin alpha 2 (Kpna2), because they are bound strongly by Pcgf6 and show expression changes with Pcgf6 depletion in ESCs. The promoter regions of Hes1, Mat2a, and Kpna2 were occupied by Pcgf6, Oct4, and TrxG components, but not by PRC1 or PRC2 complexes (Figure S3B) confirming that Pcgf6 target binding in ESCs occurs independently of PRC2 and other PRC1 complexes. Significantly, Pcgf6 was also found to be enriched at the promoters of the key transcriptional regulators in ESCs; Sox2, Oct4, Nanog, Lin28a, Lin28b, and Myc (Fig. 4B). This finding is consistent with the results of our transcriptome analysis of *Pcgf6* KD and expression patterns in Pcgf6-overexpressing ESCs (Figs 1B,C and 2B), demonstrating that Pcgf6 directly regulates the expression of these key factors. As was observed for Hes1, Mat2a, and Kpna2, the Pcgf6-bound promoter regions of the core ESC factors were also bound by Oct4 and TrxG components (Fig. 4B). Moreover, >50% of Pcgf6-bound targets were also bound by RNA polymerase II (Figure S3C), confirming the predominant association of Pcgf6 with actively transcribed genes. To further correlate the differentially expressed genes and Pcgf6-bound target genes, we compared the lists of genes from decreased genes (log_2_ < −1 at day 2 post *Pcgf6* knockdown; Table S1) and from Pcgf6-bound targets (Table S2). 368 genes are both downregulated by shRNA mediated *Pcgf6* knockdown (Fig. 1G) and bound by Pcgf6 identified by ChIP-seq (Fig. 4A). These findings reaffirm the hypothesis that Pcgf6 can serve as both a repressor and activator of bound genes. Gene ontology analysis and protein classification of these genes suggest that Pcgf6 may regulate metabolic pathways involved in stem cell pluripotency and cell reprogramming (Figure S3E,F). This is consistent with previous reports showing that genes associated with metabolic process are enriched in PRC-bound actively expressed targets^39^.

By using mass spectrometry analysis, we detected Mll1, Rbbp5 (key components of TrxG), and Zfp219 (Oct4-interacting protein^40^) co-immunoprecipitated with HA-Pcgf6 in ESCs (Table S3). Sequence analysis revealed a consensus binding sequence for Pcgf6 in ESCs (Figure S3D). PRC1 components, Rnf2 and L3mbtl2, were also co-immunoprecipitated with Pcgf6 (Figure S4A,B). Targets identified by mass spectrometry were used to generate functional protein interacting networks of Pcgf6-associated proteins using STRING (http://string-db.org). Pcgf6-associated networks show that Pcgf6 interacts with both positive (Zfp219, Mll1, Rbbp5) and repressive (Ezh1, Ezh2, Setdb1, Phc2) gene-expression regulators (Figure S4C). Among these interacting proteins, Zfp219 was reported to associate with Oct4 and was also identified in this study. To test whether Pcgf6 regulates Oct4 activity through Zfp219, we depleted *Zfp219* in ESCs and assessed the expression changes of *Oct4* mRNA. However, depletion of *Zfp219* did not affect *Oct4* or *Pcgf6* mRNA level (Figure S4D). In summary, our data provide strong evidence that Pcgf6 may function in a similar manner to TrxG, rather than PRC1, to exert its influence in maintaining ESC identities.

Finally, we asked whether the transcriptome changes observed in *Pcgf6* KD cells could be recapitulated by KD of Pcgf6-bound target genes. For this, we selected 17 Pcgf6-bound genes (top, Fig. 4C), performed specific siRNA-mediated KD in CCE ESCs, and examined changes in the expression of a panel of Pcgf6-regulated genes by RT-qPCR. Of the 17 genes examined (including *Oct4*), we found that knockdown of 14 genes faithfully reproduced the pattern of gene expression observed in Pcgf6 KD cells (yellow rectangle in Fig. 4C). In addition, knockdown of 16 of the 17 Pcgf6-bound genes caused varying degrees of reduction on *Oct4, Sox2, Klf4*, and *Nanog* expression. To further confirm Pcgf6-direct regulation of ESC key regulators, we employed two shRNAs targeting *Pcgf6* in CCE ESCs and performed ChIP-qPCR analysis on the promoter regions of Oct4 and Nanog (Figure S5A~D). With *Pcgf6* depletion by two specific shRNAs (Figure S5E), Pcgf6 ChIP-qPCR signal greatly decreased at *Oct4* promoter region by 40~50% (Figure S5A) and at *Nanog* promoter region by 50~70% (Figure S5B), compared with non-targeting shRNA-treated sample (NTC). Although the transcriptome changes dramatically with *Pcgf6* depletion in ESCs (Fig. 1), we did not detect a loss of RNA polymerase II (Pol II) binding activity at *Oct4* or *Nanog* promoter regions (Figure S5C,D), indicating that more complex modulation at transcriptional level is involved. We also examined increased expression of a few differentiation genes (*Tex11*, *Tex13*, *Mlf1*, *T*) identified by Muller and colleagues^35^. Day one post *Pcgf6* depletion in ESCs (Figure S5E), *Tex11*, *Tex13*, and *Mlf1* show minimal changes (Figure S5F, G, H) while T shows 2.5~4.5 fold increase (Figure S5I), compared with non-targeting shRNA-treated sample (NTC). The elevated expression of these differentiation genes is detected (Figure S5E, F, G, H, I) mainly at the later time points (day 7 post transfection), consistent with Muller’s finding that these genes increase slightly at day 6 post *Pcgf6* depletion in their system. Collectively, our data reveal a novel function for Pcgf6 in maintaining ESC identity by direct regulation of a number of important genes including *Kpna2, Dnajc8, Csda, Ctps, Elac2, Ivns1abp, Hes1, Cdk2, Snurf, Nudc, Nup88, Rfwd3, Rpa2, Tmpo, Mat2a,* and *Smu1* (Fig. 4D). Our data also demonstrate that Pcgf6 acts as a master regulator to positively affect the ESC core circuitry factors, including Oct4, Sox2, Nanog, and Lin28 (Fig. 4D).

## Discussion

In this study, we show that Pcgf6 is required for the expression of thousands of genes in ESCs. In addition to canonical functions of PRC1 complexes, Pcgf6 does not repress differentiation genes such as the *Hox* family, *Cbx2, Cbx4*, and ectodermal developmental regulators, but instead positively regulates the expression of genes necessary for ESC identity. *Pcgf6* expression is high in ESCs and decreases upon differentiation, and *Pcgf6* overexpression alone is able to sustain endogenous *Oct4, Sox2,* and *Nanog* expression in ESCs for more than 2 weeks (even without LIF), demonstrating the dominant role of Pcgf6 in regulating the ESC core circuitry. Finally, only a small proportion of Pcgf6 target genes are marked by H2AK119u1or bound by Rnf2, suggesting that Pcgf6 may exert atypical mechanistic roles in ESCs.

In a recent study by Zdzieblo *et al.*, Pcgf6 was identified to be a repressive transcriptional regulator, as mRNA expression of *T, Tex11, Tex13*, and *Mlf1* was elevated upon dox-induced shRNA-mediated knockdown of Pcgf6 6 days post-transduction^35^. We also assessed the mRNA level of these genes (*Tex11, Tex13, Mlf1, T*) and found consistent induction (or de-repression) at similar time points. Noticeably, down regulation of Pcgf6 lowered Oct4, Sox2 and Nanog 1 and 2 days post knockdown as shown in this study and at a later time point (day 6)^35^. Because Oct4, Sox2, and Nanog are the core transcriptional regulators in ESCs and are dysregulated following Pcgf6 knockdown, it is not possible to directly attribute transcriptional changes in the observed mesodermal-specific lineage genes and pluripotency factors to Pcgf6. Combined or in-direct transcriptional regulation by these core regulators can not be ruled out if the transcriptome changes are assessed at later time points. Therefore, the assumption that Pcgf6 acting as a repressive transcriptional regulator in ESCs may not be conclusive.

Transcriptional regulators play profound roles in modulating complex expression patterns by direct and also in-direct manners. Transcriptional changes can be detected in one or two days post induction of Ring1A/B knockout in ESCs^30^. To deconvolute the direct effects of Pcgf6 knockdown from the subsequent dysregulation in Oct4, Sox2, Nanog and other transcription factors, we analyzed transcriptome changes one and two days post-shRNA mediated knockdown in contrast to 6 days in Zdzieblo’s work^35^. We observed a consistent reduction of mRNA expression level both in day 1 and day 2 post RNAi treatment. To identify direct targets of Pcgf6 which may cause the observed transcriptomic changes and regulation of ESC identity, we utilized chromatin immunoprecipitation experiments followed by deep sequencing. The transcriptomic data, detection of TrxG components immunopreciptated with HA-tagged Pcgf6 and colocalization of chromatin binding patterns of endogenous Pcgf6, active chromatin marker H3K4me3, and the TrxG suggests a non-canonical activating role of Pcgf6 in ESCs. Our findings provide evidence of Pcgf6 immediate and direct transcriptional activities in ESCs rather than potentially indirect transcriptional regulation activities complicated by Pcgf6-mediated dysregulation of core ESC transcriptional regulators at later time points^35^. Given that the functionality of the PRC is dependent on its composition, Pcgf6-containing PRC1 may comprise two or more subsets of complexes, thus allowing it to act as both a repressor and activator. Further investigation of Pcgf6 protein interactome by employing Mass spectrometry-based immuno-precipitation proteomics is required to provide further insights into the diverse functions of Pcgf6 in ESCs.

Oct4, Sox2, and Nanog bind to their own and each other’s promoter regions, forming a positive feedback loop that secures the ESC self-renewal status. Our transcriptome analysis showed that depletion of Oct4 also decreased Pcgf6 expression in ESCs, suggesting certain indirect feedback control exist. Pcgf6 binds to the promoter regions of Hcfc1 and Ruvbl2, which have been shown to associate with TrxG complexes, showing Pcgf6 also positively regulate TrxG components^41^,^42^,^43^,^44^. Recent studies have shown that Pcgf6 is enriched in ESCs, and that *Pcgf6*-depleted ESCs show flatter cell morphology and lower alkaline phosphatase activity^33^,^34^. Collectively, these observations suggest a novel function for Pcgf6 in the positive feedback loop that maintains the ESC core identity.

Many studies have shown that the subunit composition of PRC1 complexes confers differing target gene specificities. In human 293T cells, Pcgf6 forms a PRC1.6 complex with L3mbtl2 and Wdr5^26^, and similar to our observations with Pcgf6, PRC1.6 complexes do not bind to canonical PRC1 and PRC2 targets^31^. Rather, L3mbtl2 dominantly binds to active promoters (>60% of bound genes), which is also consistent with the Pcgf6 binding pattern in our study. Wdr5, also a component of PRC1.6, has been shown to be the core component of TrxG complexes^32^, which maintain gene expression by remodeling chromatin. Together with our finding, these observations suggest that Pcgf6, Zfp219, L3mbtl2, and Wdr5 function as homeostatic regulators of transcriptional programs in ESCs by balancing the activity of repressive (PRC1) and activating (TrxG) complexes. The activating roles of Pcgf6 on transcriptional regulation are also consistent with the emerging evidence that PRC1 might be involved in transcriptional activation, together with new subsets of PRC1 complexes^45^,^46^,^47^. Indeed, the positive effect of Pcgf6 in promoting expression of Oct4, Sox2, and Nanog is likely supported by the binding of TrxG complexes and maintenance of an open chromatin conformation. Furthermore, among Pcgf6-bound targets, genes associated with metabolic processes, known to be critical for maintaining self-renewal and determining cell fate^48^,^49^, are highly enriched. This finding is consistent with PRC-bound actively expressed genes reported in a previous study^39^. Moreover, the activating role of Pcgf6 in this study may explain why many PRC-bound genes (1227 genes) are actively expressed in ESCs^39^. Interestingly, Pcgf6 is known to be phosphorylated along with cell cycle^41^, suggesting a possible mechanism by which Pcgf6 could interact with other transcriptional activators. A detailed biochemical analysis of Pcgf6-containing PRC1 complexes in ESCs is required to understand the mechanism of various components of PRC1 complexes in directing specific functions. Future studies of how Pcgf6 phosphorylation influences the protein interactome will undoubtedly provide insight into the mechanisms underlying Pcgf6 regulation of ESC-specific gene expression.

## Methods

### Reprogramming of Oct4-EGFP MEFs and Derivation of Mouse Pcgf6-iPSCs

Induced reprogramming of MEFs was conducted as described^50^. In brief, Oct4-EGFP MEFs were plated in 12-well plates at 4 × 10^4^/well and transduced for 3 days with pMXs retroviruses expressing Oct4, Sox2, Klf4, and c-Myc (OSKM; plasmids from Addgene). Cells were then transferred to mouse ESC medium (DMEM with 15% fetal bovine serum, nonessential amino acids, L-glutamine, monothioglycerol, and 1000 U/ml LIF) for the remainder of the reprogramming process. The medium was exchanged every other day. After ~2 weeks, the cultures were examined by fluorescence microscopy, and fully reprogrammed cells (referred to as Oct4-EGFP-miPSCs) were scored as EGFP+ colonies. Pcgf6-miPSCs were derived as described above except a *Pcgf6*-encoding retrovirus was added at the same time as the OSKM retroviruses. EGFP+ colonies were scored as above, and individual colonies were manually selected under a stereo microscope (Leica). The colonies were dissociated with 0.05% trypsin with EDTA for 5–15 min and then re-seeded on 0.1% gelatin-coated tissue culture plates in ESC medium.

### Reprogramming of Human BJ Fibroblasts and Derivation of Human Pcgf6-iPSCs

All work was approved by the Institutional Review Board at Sanford Burnham Prebys Medical Discovery Institute. Human BJ fibroblasts obtained from ATCC were placed in 12-well plates at 5.5 × 10^4^/well, transduced with OSKM pMXs retroviruses for 3 days, and then re-seeded at a density of ~5 × 10^4^ cells/well in 6-well plates containing a layer of irradiated MEF feeder cells. The next day, cells were transferred to human ESC medium (details in Supplemental Information) and the medium was subsequently changed daily. After 3–4 weeks, reprogramming efficiency was assessed by alkaline phosphatase (AP) staining. hiPSCs overexpressing Pcgf6 (Pcgf6-hiPSCs) were derived in the same way except a *Pcgf6*-encoding retrovirus was added at the same time as the OSKM retroviruses. Three weeks post-transduction, ESC-like colonies were manually selected and cultured on a layer of irradiated MEFs for several passages before analysis.

### Teratoma Formation by Mouse and Human iPSCs

All animal work was approved by the Institutional Review Board at Sanford Burnham Prebys Medical Discovery Institute and was performed in accordance with Institutional Animal Care and Use Committee guidelines. miPSCs were resuspended in mouse ESC medium at 10^7^/ml, and 150 μl was injected subcutaneously into the dorsal side of the hind legs of anesthetized athymic nude mice. Mice were sacrificed 3–4 weeks later and tumors were collected, fixed, sectioned, stained, and analyzed in the Sanford-Burnham Medical Research Institute (SBMRI) Cell Imaging and Histology core facility. For human cell-derived teratomas, hiPSCs were grown to ~50% confluency in 10-cm culture dishes and then harvested by collagenase treatment. Cells were resuspended in human ESC medium containing 40% Matrigel (BD Biosciences, 356231) and clumped by low-speed centrifugation. Aliquots (~50 μl) of cell clumps were injected under the kidney capsule of anesthetized SCID mice. Mice were sacrificed at 10–12 weeks after injection, and the tumors were collected and analyzed as described above.

### Chromatin Immunoprecipitation and High-Throughput Sequencing

CCE cells were cross-linked with disuccinimidyl glutarate and paraformaldehyde, and nuclear extracts were prepared using cell extraction buffers^51^. ChIP-seq analysis was conducted as described^51^ using two independent anti-Pcgf6 antibodies (Santa Cruz, sc-160649; Abcam, ab48010). After pull-down, genomic DNA was extracted and amplified using an Illumina Library Prep Kit (Illumina, IP-202-1012), and the DNA was subjected to next-generation high-throughput sequencing analysis. ChIP-seq data peaks were analyzed and identified (cutoff *p*-value 0.00001) using MACS^52^. ChIP-seq peaks residing in the promoters (specified as ±10 kb of transcription start sites [TSSs]) of mm9 RefSeq genes were annotated by CEAS^53^.

### Microarray Analysis

CCE ESCs were transfected with control non-targeting siRNA or siRNA against *Pcgf6* or *Oct4* for 24 or 48 h, and total RNA was then isolated using TRIzol (Invitrogen). Quality of total RNAs were examined by Bioanalyzer (Agilent) before hybridization of microarray analysis. MouseWG-6 v2.0 Expression BeadChip (illumina) kits were used to detect total RNA expression profiling. Sample-independent spiked oligonucleotides were used during hybridization to monitor data quality and provide proper controls (http://res.illumina.com/documents/products/technotes/technote_gene_expression_data_quality_control.pdf). Data of gene expression profiling was analyzed in the SBMRI Genomics and Bioinformatics core facilities. Gene clusters were created using Cluster 3.0, heat maps were created using Java TreeView, and scatter plots were created using Excel.

### Meta-Analysis Using IPA and NextBio Platforms

To analyze transcriptome changes, normalized mRNA expression data were uploaded onto the IPA server. For *Pcgf6*- or *Oct4*-depleted ESCs, the cut off value for fold-change and *p* (Fishers’ Exact test) were 1.5 (±0.6 log_2_ scale) and 0.05, respectively. Changes in representation of genes, networks, and pathways were identified using the IPA platform.

## Additional Information

**How to cite this article**: Yang, C.-S. *et al.* Polycomb Group Protein Pcgf6 Acts as a Master Regulator to Maintain Embryonic Stem Cell Identity. *Sci. Rep.* **6**, 26899; doi: 10.1038/srep26899 (2016).

## Supplementary Material

Supplementary Information

Supplementary Table 1

Supplementary Table 2

Supplementary Table 3
