# Supplementary material for: Polycomb Group Protein Pcgf6 Acts as a Master Regulator to Maintain Embryonic Stem Cell Identity
Source: Sci Rep. 2016 Jun 1;6:26899. doi: 10.1038/srep26899 (PMC4888081; doi:10.1038/srep26899)
Supplement: Supplementary Information [file srep26899-s1.pdf]

SUPPLEMENTARY INFORMATION

Polycomb Group Protein Pcgf6 Acts as a Master Regulator to Maintain Embryonic Stem Cell Identity

Chao-Shun Yang,[1] Kung-Yen Chang,[1] Jason Dang, and Tariq M. Rana[1,2*]

[1]Department of Pediatrics, University of California San Diego School of Medicine, 9500 Gilman Drive MC 0762, La Jolla, California 92093

[2]Institute for Genomic Medicine and Moores Cancer Center, University of California San Diego School of Medicine, 9500 Gilman Drive, La Jolla, California 92093

- Correspondence: trana@ucsd.edu

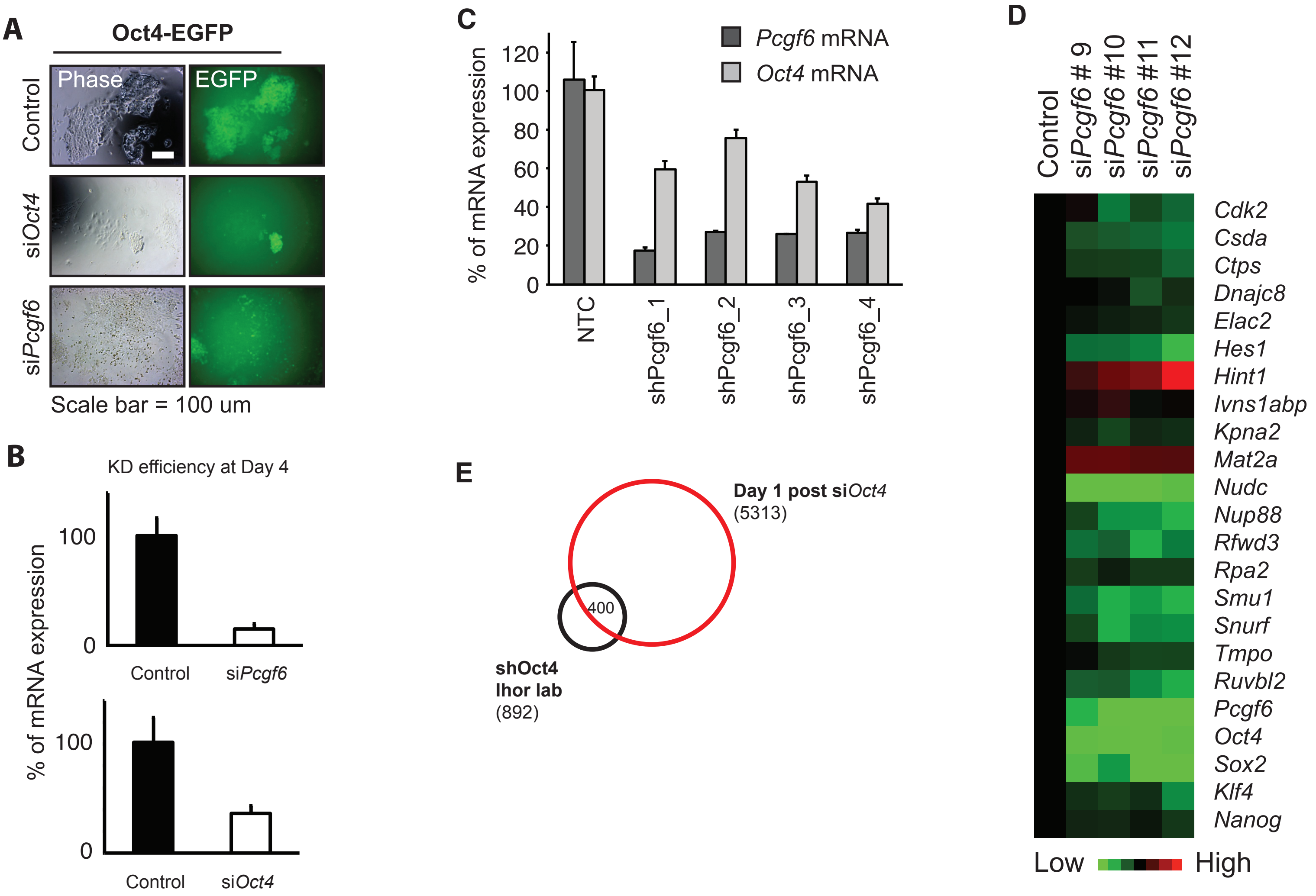

F

	Annotation Clusters	*p* value
siOct4	Organelle part	1.40E-40
	Intracellular non-membrane-bounded organelle	1.20E-20
	Mitochondrion	4.50E-20
	Chromosome	5.60E-14
	Nucleotide binding	2.20E-16
	Ribonucleoprotein complex	4.90E-18
	RNA processing	8.00E-13
	DNA metabolic process	2.10E-09
	Protein transport	1.20E-09
	Cell cycle	1.10E-10
shOct4_Ihor lab	Non-membrane-bounded organelle	1.90E-19
	Cytoskeletal protein binding	9.40E-08
	Blood vessel morphogenesis	6.30E-06
	Basolateral plasma membrane	5.70E-07
	Programmed cell death	1.20E-04
	Cell cycle	7.10E-08
	Contractile fiber part	2.70E-05
	Cell migration	1.40E-04
	In utero embryonic development	1.40E-05
	Nucleotide binding	4.00E-05

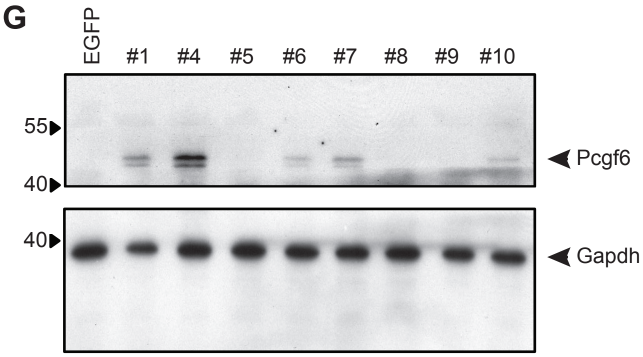

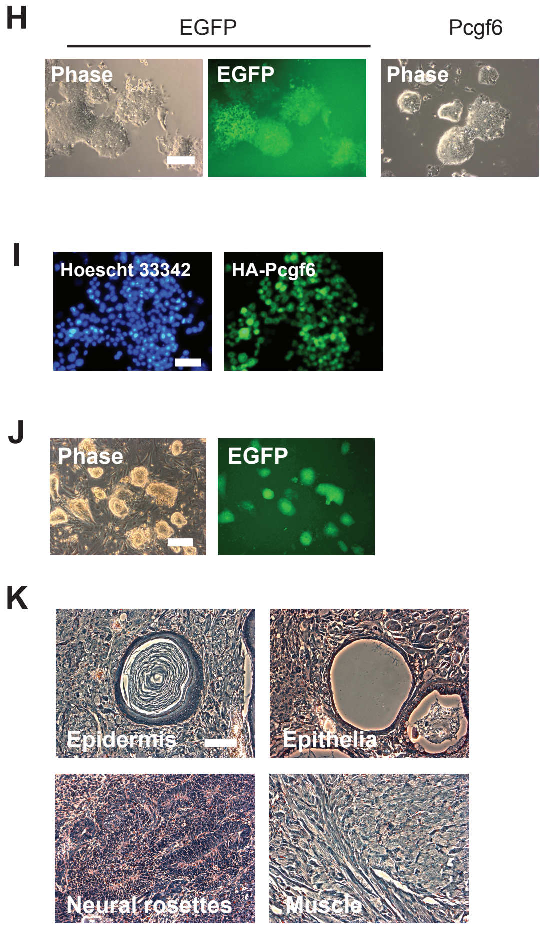

Figure S1.

Pcgf6 play important roles to maintain ESC properties and to enhance iPSC generation

(A) Phase contrast and fluorescence micrographs of reprogrammed Oct4-EGFP-miPSCs 4 days after transfection with non-targeting (Control), Oct4, or Pcgf6 siRNAs for 3–5 h. Scale bar = 100 μm.

(B) Bar graph showing mRNA KD by siRNA transfection in CCE ESCs at day 4 post siRNA transfection. CCE ESCs were transfected with siRNA smart pools (as indicated) targeting either Pcgf6 or Oct4 and total RNAs were extracted four days later. Oct4 and Pcgf6 gene expression was detected by RT-qPCR. Relative expression ratio was calculated by normalized to expression level of non-targeting siRNA-treated cells (Control). Error bars denote standard errors of two independent experiments.

(C) Bar graph showing decreased Oct4 gene expression after Pcgf6 KD by four distinct pLKO shRNAs. CCE ESCs were transfected with four distinct shRNAs (as indicated) and total RNAs were extracted two days later. Oct4 and Pcgf6 gene expression was detected by RT-qPCR. Relative expression ratio was calculated by normalized to expression level of non-targeting shRNA-treated cells (NTC). Error bars denote standard errors of two independent experiments.

(D) Confirmation of Pcgf6 and target gene downregulation with 4 distinct siRNAs. Heat map showing mRNA expression profiles of CCE ESCs transfected with non-targeting siRNA (Control) or 4 different siRNAs targeting Pcgf6. Expression of the indicated genes (including Pcgf6) was analyzed by RT-qPCR at 24 hours post-transfection, and the results are expressed relative to levels in CCE ESCs transfected with control siRNA.

(E) Venn diagram showing overlapped differentially expressed genes with Oct4 depletion in ESCs. Total RNAs were isolated from CCE ESCs one day post siOct4 transfection and used to perform microarray analysis. Differentially expressed genes (log_2 > +/- 0.6) were dientified and compared with data from Ihor lab (Ang et al., 2011). The number of differentially expressed genes of each treatment are shown in parenthesis and overlapped genes shown in the center of diagram.

(F) GO analysis (DAVID) showing functional annotation clusters of differentially expressed genes with Oct4 depletion in ESCs.

(G) Western blot analysis of CCE ESC clones overexpressing Pcgf6.
Eight stable HA-Pcgf6 transfectants were analyzed 3 weeks after selection in G418, and clones 1, 4, 6, and 7 were used in the experiments described here. CCE ESCs expressing EGFP served as control. Gapdh served as a protein loading control.

(H) Phase contrast, fluorescence, and light microscopy images showing normal morphology of a Pcgf6-mESC clone compared with control EGFP-expressing CCE ESCs. One representative clone of each is shown. Scale bar = 100 μm.

(I) Fluorescence microscopy images showing correct nuclear localization of Pcgf6 transgene in OSKM-reprogrammed Oct4-EGFP MEFs. HA-tagged Pcgf6 expression was detected 5 days post-OSKM transduction by immunostaining with anti-HA primary and Alexa Fluor 488-tagged secondary antibody. Nuclei were stained with Hoescht 33342. Scale bar = 25 μm.

(J) Phase contrast and fluorescence microscopy images showing normal ESC morphology of miPSCs derived with OSKM and Pcgf6. Oct4-EGFP MEFs were used for iPSC generation. Activation of endogenous Oct4 is indicated by EGFP fluorescence. Scale bar = 200 μm.

(K) Sections from a teratoma derived from Pcgf6-miPSCs. Reprogrammed cells were injected subcutaneously into the dorsal hind legs of nude mice and tumors were collected at ~3 weeks post-injection. The indicated tissue types were identified by histopathological analysis. Scale bar = 50 μm.

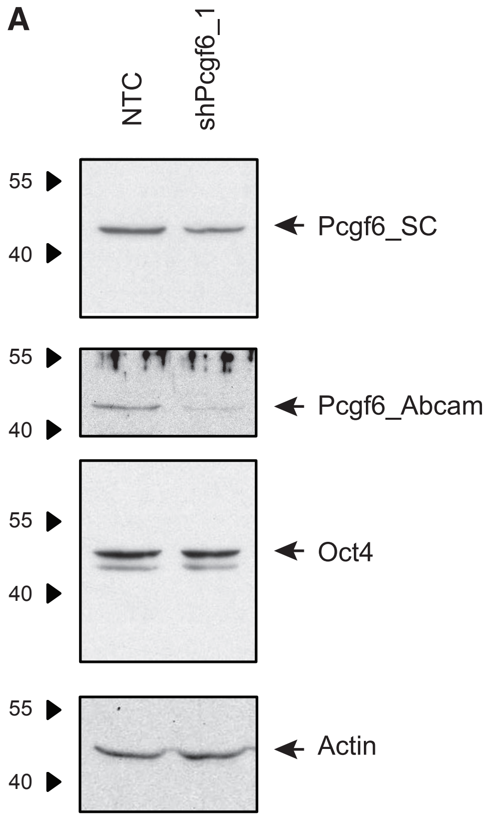

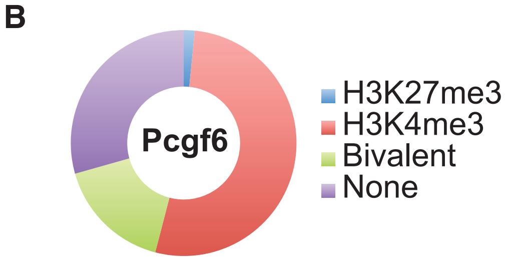

C

	Pcgf6	L3mbtl2	Suz12	Eed	Phc1	Rnf2
H3K27me3	2.1%	11.1%	19.8%	15.8%	24.9%	33.5%
H3K4me3	**74.7%**	**62%**	2.7%	2.9%	1.6%	2.2%
Bivalent	23.2%	26.9%	**61.9%**	**68.2%**	**58.3%**	**48.7%**

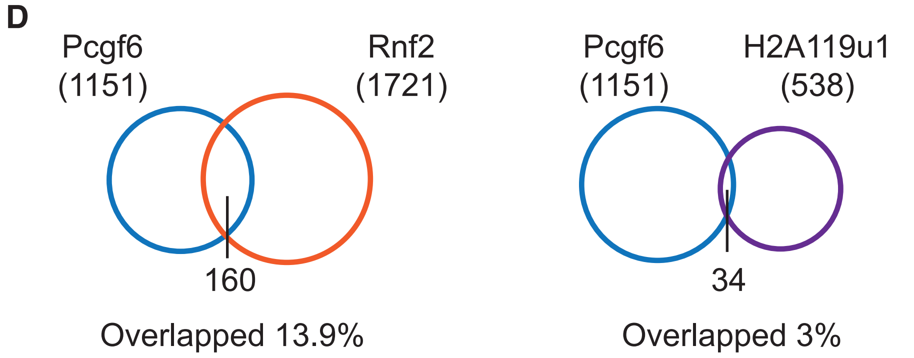

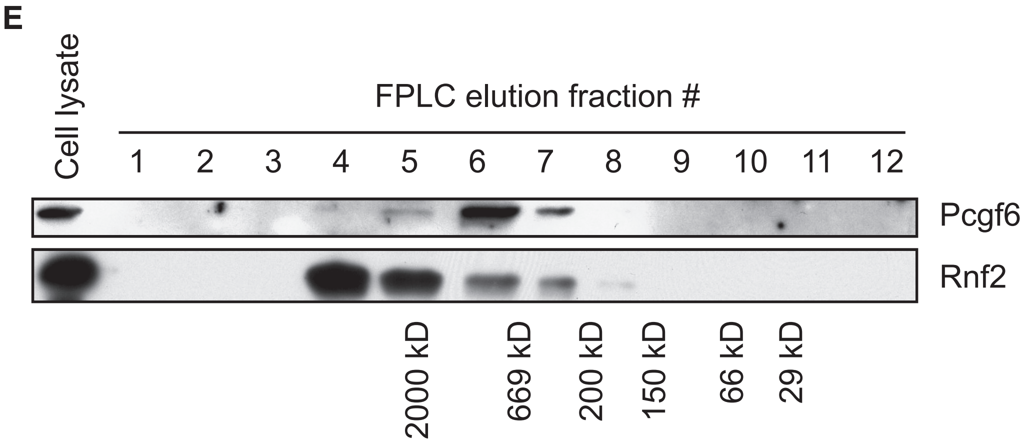

Figure S2.

Atypical Function of Pcgf6 in mESCs

(A) Western blot analysis showing specificity of Pcgf6 antibodies used for the following chromatin immunoprecipitation assays. CCE ESCs were transfected with pLKO shRNAs targeting Pcgf6 and cell lysate was isolated ~24 hours later. Pcgf6 proteins were detected with two different antibodies (Santa Cruz, sc-160649; Abcam, ab48010). Oct4 proteins were detected with specific antibody (Stemgent, 09-0023). Actin served as a protein loading control.

(B) Pie chart of Pcgf6-bound promoters showing that the majority are decorated with active epigenetic marks in CCE ESCs. Pcgf6-bound targets were compared with published data of H3K4me3-bound (active) and H3K27me3-bound (repressed) targets in mouse ESCs (Mikkelsen et al., 2007). Bivalent genes are marked by both H3K4me3 and H3K27me3 and are considered poised/repressed.

(C) Quantification of ChIP-seq data showing the percentage of promoters bound by Pcgf6 (this study), L3mbtl, Suz12, Eed, Phc1, Rnf2 (Boyer et al., 2006; Ku et al., 2008; Marson et al., 2008; Mikkelsen et al., 2007; Qin et al., 2012) that also contain histone modifications.

(D) Venn diagram showing low overlap between genomic loci bound by Pcgf6 and those bound by Rnf2 (canonical PRC1 complexes) in CCE ESCs. ChIP-seq-identified gene targets bound by Pcgf6 were annotated with promoter regions ± 3kb of the TSSs. Published ChIP-chip data were used for Rnf2 and H2A119u1 (Endoh et al., 2012). The number and percentage of overlapping genes is indicated.

(E) Western blot analysis of FPLC fractions from CCE ESCs showing separation of Pcgf6- and Rnf2-containing canonical PRC1 complexes. Total cell lysate was probed as a positive control. Apparent molecular weights are indicated.

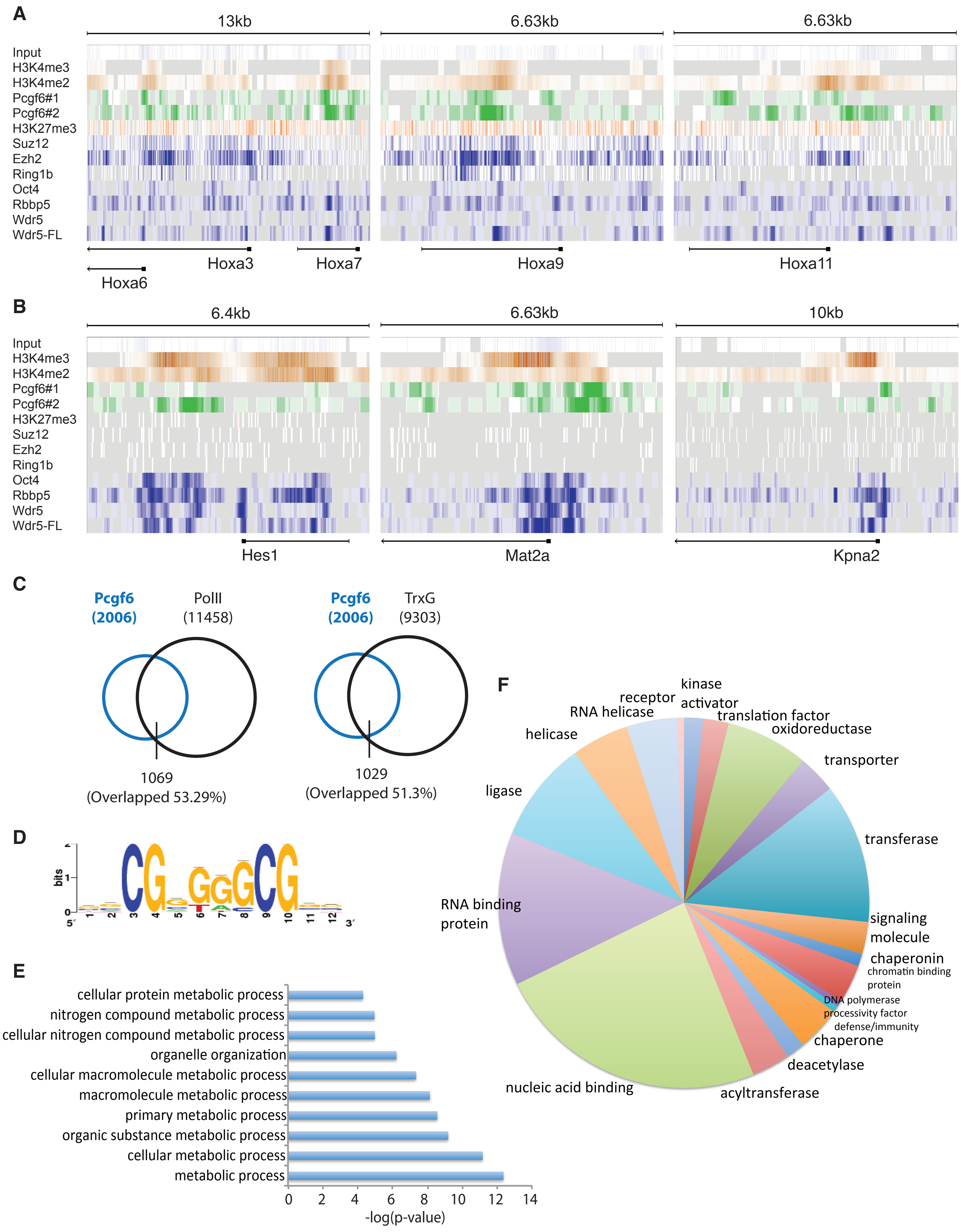

A
13kb
6.63kb
6.63kb
Input
H3K4me3
H3K4me2
Pcgf6#1
Pcgf6#2
H3K27me3
Suz12
Ezh2
Ring1b
Oct4
Rbbp5
Wdr5
Wdr5-FL
Hoxa3
Hoxa7
Hoxa9
Hoxa11
Hoxa6
B
6.4kb
6.63kb
10kb
Hes1
Mat2a
Kpna2
C
Pcgf6 (2006)
PolII (11458)
1069 (Overlapped 53.29%)
Pcgf6 (2006)
TrxG (9303)
1029 (Overlapped 51.3%)
D
bits
5′
3′
E
cellular protein metabolic process
nitrogen compound metabolic process
cellular nitrogen compound metabolic process
organelle organization
cellular macromolecule metabolic process
macromolecule metabolic process
primary metabolic process
organic substance metabolic process
cellular metabolic process
metabolic process
0 2 4 6 8 10 12 14
-log(p-value)
F
kinase
receptor
activator
RNA helicase
translation factor
oxidoreductase
helicase
transporter
ligase
transferase
RNA binding protein
signaling molecule
chaperonin
chromatin binding protein
DNA polymerase processivity factor
defense/immunity
chaperone
deacetylase
nucleic acid binding
acyltransferase

Figure S3.

Pcgf6 Function is Atypical Compared with Canonical PRC1 Complexes

(A) Pcgf6 binds to the Hox gene cluster. IGV analysis was conducted as described for Figure 4B. The scale of each genomic region is indicated on the top of each panel. TSSs of the target genes are shown as black squares and the solid line indicates the transcription direction. The color code of bound promoters regions is: green = Pcgf6; orange = H3K4me2, H3K4me3, and H3K27me3; and blue = regions bound by Oct4, PRC1, PRC2, and TrxG components.

(B) Pcgf6-bound promoters are also bound by TrxG components, but not by PRC1 or PRC2 components. IGV analysis was conducted as described above. The color codes are as in (A).

(C) Venn diagram showing that >50% of Pcgf6-bound targets are also bound by positive regulators (RNA polymerase II and TrxG proteins) in ESCs. ChIP-seq data for TrxG proteins were obtained from the Ihor lab (Ang et al., 2011) and data for PolII were from the Ren lab (Shen et al., 2012). The number and percentage of overlapping genes is indicated.

(D) Consensus sequence of Pcgf6-binding sites in ESCs. Peaks of Pcgf6-bound regions were analyzed by MACS. Annotated Pcgf6-bound regions within 3kb up- and down-stream to transcription start sites (TSS) were analyzed to extract genomic DNA sequences. Motif discovery was performed and visualized by using RSAT (Shin et al., 2009).

(E) Gene ontology of PCGF6 bound genes which are also differentially expressed when PCGF6 is knocked down by shRNA.

(F) Protein classification of PCGF6 bound genes which are also differentially expressed when PCGF6 is knocked down by shRNA.

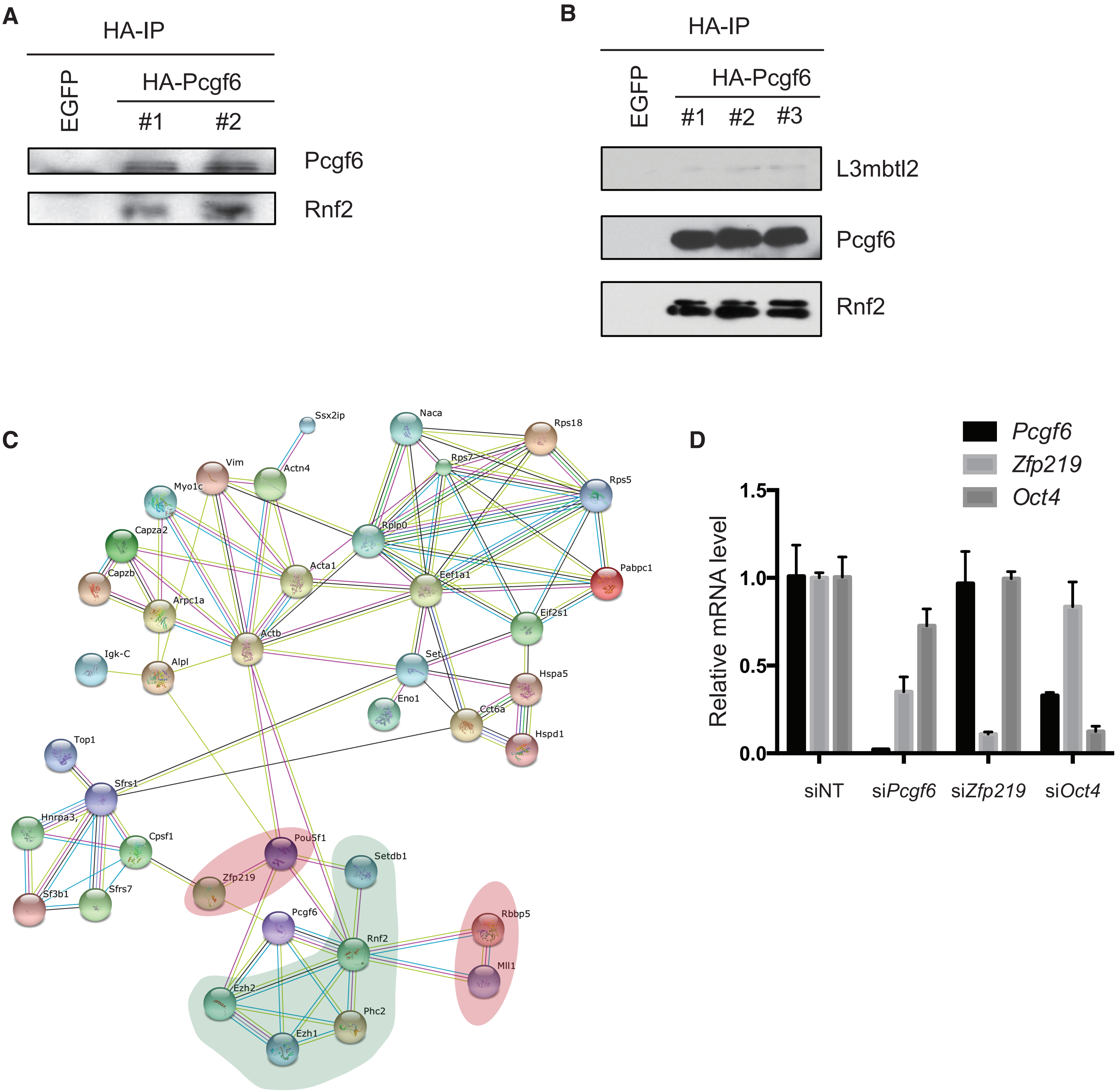
A
HA-IP
EGFP
HA-Pcgf6
#1
#2
Pcgf6
Rnf2
B
HA-IP
EGFP
HA-Pcgf6
#1
#2
#3
L3mbtl2
Pcgf6
Rnf2
C
Ssx2ip
Naca
Rps18
Rps7
Rps5
Vim
Actn4
Myo1c
Rplp0
Capza2
Capzb
Acta1
Eef1a1
Pabpc1
Arpc1a
Eif2s1
Actb
Igk-C
Alpl
Set
Hspa5
Eno1
Cct6a
Hspd1
Top1
Sfrs1
Hnrpa3
Cpsf1
Pou5f1
Setdb1
Zfp219
Sf3b1
Sfrs7
Pcgf6
Rbbp5
Rnf2
Mll1
Ezh2
Phc2
Ezh1
D
Pcgf6
Zfp219
Oct4
Relative mRNA level
1.5
1.0
0.5
0.0
siNT
siPcgf6
siZfp219
siOct4

Figure S4.

Pcgf6 Interacts with Various Protein Complexes

(A) and **(B)** Western blotting analysis showing that Pcgf6 interact with PRC1 complexes.
CCE ESCs (A) or 293FT (B) were transfected with pcDNA-HA-Pcgf6 or pcDNA-EGFP. Two days later, nuclear extract was prepared using CelLytic NuCLEAR extraction kit and Pierce IP lysis buffer. Magnetic beads conjugated with anti-HA antibodies were used to immunoprecipitate HA-Pcgf6 and associated proteins. IP-pull down proteins were resolved by SDS-PAGE and detected with specific antibodies against endogenous proteins. Cell lysate of EGFP-transfected cells was used as negative control. Biological repeats were indicated as #1, #2, or #3.

(C) STRING protein interaction analysis showing Pcgf6 interacts with activating and repressive complexes. Pcgf6-associated proteins were idnetified with co-IP and mass spectrometry (Supplemental File 3). Interaction of Pcgf6-associated proteins was analyzed using STRING database (http://string-db.org/). Components of activating complexes were identified (Zfp219, Rbbp5, Mll1; highlighted in red), while repressive-complex components were also identified (Setdb1, Phc2, Ezh1, Ezh2; highlighted in green).

(D) Bar graphs showing relative mRNA expression level in CCE ESCs treated with various siRNAs. Cells were transfected with various siRNAs as indicated and total RNAs were isolated for performing RT-qPCR analysis. Relative mRNA expression level was calculated by normalizing to control (siNT). Error bars denote standard errors of two independent experiments.

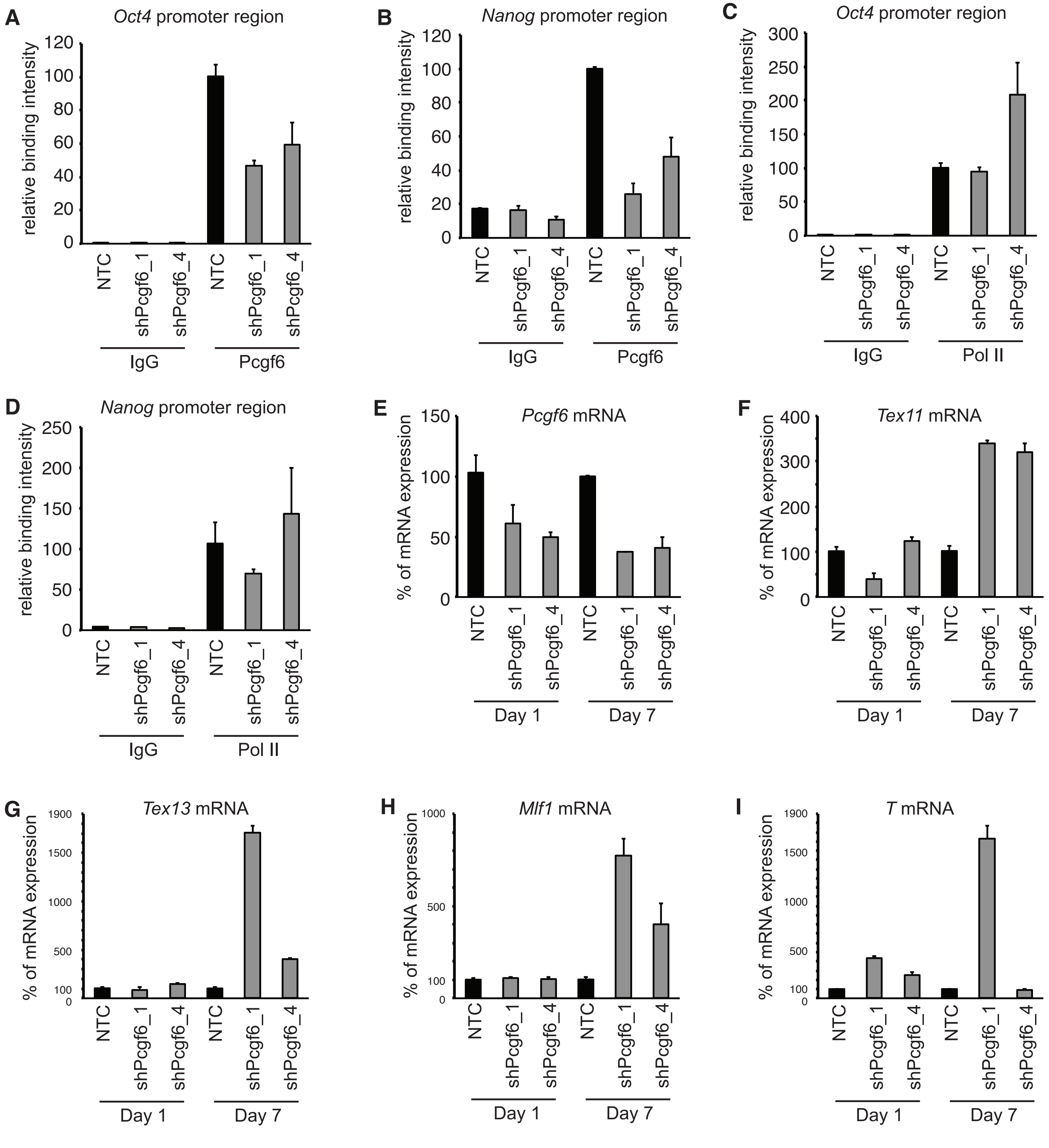

A
Oct4 promoter region
relative binding intensity
NTC
shPcgf6_1
shPcgf6_4
IgG
Pcgf6
B
Nanog promoter region
C
Oct4 promoter region
Pol II
D
Nanog promoter region
E
Pcgf6 mRNA
% of mRNA expression
Day 1
Day 7
F
Tex11 mRNA
G
Tex13 mRNA
H
Mlf1 mRNA
I
T mRNA

Figure S5.

Direct Binding of Pcgf6 on Oct4 and Nanog Promoter Regions Verified by ChIP-qPCR.

(A) to (D) Bar graphs showing binding intensity of Pcgf6 and RNA polymerase II (Pol II) on Oct4 and Nanog regions. CCE ESCs were transfected with two distinct pLKO shRNAs targeting Pcgf6. Two days post transfection, cells were fixed and crosslinked with DSG and paraformaldehyde as described in Experimental Procedures. Non-targeting shRNA served as negative control (NTC; black bars) for transfection. Chromatins were prepared and immunoprecipitation was performed by using specific Pcgf6 or Pol II antibodies. IgG antibody served as negative control (IgG) for ChIP assays. Co-precipitated chromatins were extracted by phenol/chloroform/isoamyl alcohol to isolate DNAs. Binding intensities were detected by RT-qPCR and relative ratio was calculated by normalizing to input signal. Error bars denote standard errors of two wells.

(E) to (I) Bar graphs showing increase of differentiated gene expression at 7 days after Pcgf6 depletion in ESCs. CCE ESCs were transfected with two distinct pLKO shRNAs targeting Pcgf6 as indicated. Cells were harvested at 1 or 7 days post transfection. Transfected cells were selected with puromycin (2 ng/ml) for 7-day culture condition to maintain shRNA expression. Total RNAs were isolated with TRIZOL reagent and RT-qPCR was performed to detect mRNA expression of select genes as indicated. Relative ratio of mRNA expression was calculated by normalizing to non-targeting control (NTC; black bars). Error bars denote standard errors of two independent experiments.

SUPPLEMENTARY EXPERIMENTAL PROCEDURES

Oct4-EGFP Mouse Embryonic Fibroblasts

Oct4-EGFP MEFs were derived from the mouse strain B6;129S4-Pou5f1$^{tm2(EGFP)Jae}$/J (Jackson Laboratory; stock #008214) [53] using the protocol provided on the WiCell Research Institute website (http://www.wicell.org/). In brief, E13.5 embryos were collected from time-mated pregnant female mice and MEF cells were isolated by mincing and trysinizing embryos. Oct4-EGFP MEFs were maintained in MEF complete medium (DMEM with 10% FBS, nonessential amino acids, L-glutamine, in the absence of sodium pyruvate). Robustly growing cells (usually <4 passages) were used for induced reprogramming experiments.

Construction of Retroviral Vectors Expressing Transgenes

Pcgf6 cDNA was obtained by reverse transcription using total RNA from CCE ESCs and inserted into the retroviral vector pMXs downstream of the HA sequence (Addgene; http://www.addgene.org/). Ecotropic retroviruses expressing the transgenes were produced in Plat-E cells (Cell Biolabs, RV-101) and amphotropic viruses were produced in Plat-A cells (Cell Biolabs, RV-102) for transduction of mouse or human somatic cells, respectively. Virus-containing supernatants were collected 2 days post-transfection and added to cells with 6 μg/ml of polybrene (Millipore) to enhance transduction efficiency.

Human Induced Pluripotent Stem Cell Culture

Derived human iPSCs were cultured following the protocols available on the WiCell Research Institute website (http://www.wicell.org/). Briefly, cells were grown in DMEM/F12 medium (Invitrogen, 11330-032) containing 20% Knockout serum replacer (Invitrogen, 10828), 4 ng/ml human recombinant basic fibroblast growth factor (bFGF; Invitrogen, 13256-029), 1 mM L-glutamine (Invitrogen, 25030081), and 1% nonessential amino acids (Invitrogen; Cat# 11140-050). iPSCs were seeded on a feeder layer of MEFs at early passages (1 to 4). MEFs were irradiated and seeded at 1.88×10^5 cells per well in a six-well plate. For long-term cultures, hESC medium was conditioned by incubation with MEFs (2.12×10^5 cells/ml) for 24 h, and bFGF was added before use.

Immunofluorescence and Alkaline Phosphatase Staining

Human iPSCs were analyzed for pluripotency markers as described [54]. Briefly, BJ-derived hiPSCs were fixed with 4% paraformaldehyde (Electron Microscopy Sciences, 15710-S) in PBS for 30 min at room temperature (RT), and permeabilized for 5 min at RT by treating with 0.1% Triton X-100 in PBS. Permeabilized cells were blocked with 5% goat serum in PBS for 30 min at RT and then incubated for 1 h at RT with antibodies to SSEA-4 (Santa Cruz, sc-21704), Tra-1-60 (Cell Signaling, 4746), Tra-1-81 (Cell Signaling, 4745), and Nanog (R&D Systems, AF1997) diluted at 1:500 in 1.5% goat serum in PBS. Cells were washed 3 times with PBS and then incubated for 1 h at RT with secondary antibodies (Alexa Fluor 488- or 546-conjugated) diluted 1:200 in 1.5% goat serum in PBS. Cell nuclei were stained with DAPI (4′,6 diamidino-2-phenylindole). Fluorescence images were captured by on a Leica fluorescence microscope

equipped with a charge-coupled device camera. AP staining of fixed cells was performed with a Vector Red Alkaline Phosphatase Substrate Kit I (Vector Laboratories, SK-5100) according to the manufacturer's instructions.

Karyotyping of Human iPSCs

The karyotypic integrity of hiPSCs was assessed by cytogenetic analysis (performed by Applied StemCell, Inc). In brief, Pcgf6-hiPSCs were treated overnight with colcemid and dissociated for G-band staining and chromosome counting. Chromosomes in the metaphase of 20 cells were counted, and 4 cells were examined for G-band staining with band resolution at 450–525.

Derivation of Pcgf6-mESCs

HA-tagged *Pcgf6* cDNA was cloned into pcDNA and transfected into CCE ESCs. Control cells were transfected with pcDNA-EGFP. Transfected cells were treated with geneticin (G418) for at least 3 weeks before stable clones were manually selected. A total of 8 clones were examined for Pcgf6 expression by western blotting. Of the 5 positive clones, 4 with the highest Pcgf6 expression were selected for *in vitro* differentiation assays (#1, #4, #6, and #7).

***In vitro* Differentiation of Pcgf6-mESCs**

Pcgf6-mESCs generated as described above were dissociated by trypsin/EDTA treatment and resuspended in embryoid body (EB) medium (DMEM with 15% FBS, 1X nonessential amino acids, and 2 mM L-glutamine) at a final concentration of 5×10^4 cells/ml. EB formation was

induced by dispensing 20 µl (1000 cells) aliquots in hanging drops on an inverted Petri dish lid. Three days later, the EBs were collected and transferred to 0.1% gelatin-coated 6-well plates at 10–20 EBs/well. The colonies were observed on an inverted microscope every 3 days for 15 days. Cells were also collected for extraction of total RNA at the same time points (days 0, 3, 6, 9, 12, 15).

Western Blot Analysis

Pcgf6-mESCs and Pcgf6-hiPSCs were lysed in M-PER buffer (Pierce), centrifuged, and the supernatants removed. Equal amounts of total protein were separated by 10% SDS-PAGE and transferred to PVDF membranes for western blotting with an anti-HA antibody (Roche Applied Science, 1186742300). GAPDH (Santa Cruz, sc-20357) and β-actin (Thermo Scientific, MS-1295) served as loading controls. Target proteins were detected with SuperSignal West Femto Chemiluminescent Substrate (Thermo Scientific, 34094).

siRNA-Mediated Gene Knockdown for Testing Reprogramming Efficiency

Specific siRNAs against *Pcgf6* were purchased from Dharmacon (Thermo Scientific). Oct4-EGFP MEFs were transfected with a mixture of siRNA and Lipofectamine (Invitrogen) according to the manufacturer's instructions. Typically, at least 25 nM (final concentration) of each siRNA was used to effectively deplete Pcgf6. After 3–5 h, the transfection reagent was discarded and OSKM retrovirus-containing supernatant was added to induce reprogramming. Colonies were analyzed for EGFP expression 2 weeks later. Gene knockdown efficiency was

evaluated by semi-quantitative real-time RT-PCR ~24 h after retroviral transduction (data not shown). *Gapdh* mRNA served as an internal control to normalize mRNA expression signals.

siRNA-Mediated Gene Knockdown for Testing ESC Identity

Reprogrammed Oct4-EGFP-miPSCs or CCE ESCs were seeded at 1×10^5 cells/well in 12-well plates coated with 0.1% gelatin. The next day, cells were transfected by the addition of lipofectamine/siRNA complexes (25–50 nM final concentration) for 3–5 h, after which the transfection reagent was discarded and mESC medium was added. Four days after transfection, EGFP was detected by fluorescence microscopy or cells were stained for AP and examined by light microscopy.

Heatmap Visualization of ChIP-Seq Data Sets

ChIP-Seq data sets for H3K4me3, H3K27me3 (GSE12241) [35], Rnf2, Ezh2, Suz12 (GSE13084) [36], Rbbp5, and Wdr5 (GSE22934) [32] in mESCs were obtained from the Gene Expression Omnibus (GEO). Data files with mm8 coordinates were converted to mm9 using UCSC liftOver tool. The genomic regions 2 kb upstream and downstream of the TSSs of H3K4me3- and H3K27me3-binding genes were divided into 100-bp bins. Regions flanking the TSSs of the same genes were used for detection of PRC1, PRC2, and TrxG protein binding. For each bin, the number of overlapping reads was calculated and normalized to the total reads. The normalized read densities of ChIP-seq signals were visualized as heat maps using Java TreeView.

Motif Analysis of Pcgf6 Consensus Binding Sites

Peaks of ChIP-seq data were identified using MACS, with a *p*-value cutoff of 0.00001 [49]. With promoter regions defined as 3 kb upstream and downstream of TSSs, the ChIP-seq regions residing in the promoters of mm9 RefSeq genes were annotated by CEAS [50]. The results obtained from ChIP-seq with 2 different Pcgf6 antibodies were merged, and the redundant peaks were removed. FASTA sequences of 1230 resulting ChIP-seq peaks were extracted from genomic coordinates. Motif discovery for Pcgf6 was performed by RSAT [50].
